# A Review of Security Evaluation of Practical Quantum Key Distribution System

**DOI:** 10.3390/e24020260

**Published:** 2022-02-10

**Authors:** Shihai Sun, Anqi Huang

**Affiliations:** 1 School of Electronics and Communication Engineering, Sun Yat-Sen University, Shenzhen 518107, China; 2 Institute for Quantum Information & State Key Laboratory of High Performance Computing, College of Computer Science and Technology, National University of Defense Technology, Changsha 410073, China

**Keywords:** quantum cryptography, quantum communication, quantum key distribution, practical security, security evaluation

## Abstract

Although the unconditional security of quantum key distribution (QKD) has been widely studied, the imperfections of the practical devices leave potential loopholes for Eve to spy the final key. Thus, how to evaluate the security of QKD with realistic devices is always an interesting and opening question. In this paper, we briefly review the development of quantum hacking and security evaluation technology for a practical decoy state BB84 QKD system. The security requirement and parameters in each module (source, encoder, decoder and detector) are discussed, and the relationship between quantum hacking and security parameter are also shown.

## 1. Motivation

Quantum key distribution (QKD) provides an approach to share a *key* between two remote parties via an insecure channel with information-theoretic security (or called the unconditional security). Since the first QKD protocol, BB84, was proposed by Bennett and Brassard in 1983 [1], various types of QKD protocols based on the discrete variables [2,3,4] or the continuous variables [5,6] have been proposed, which have been applied to different situations according to their characteristics. Remarkably, QKD-based quantum networks are also available in many countries [7,8,9]. For example, an integrated space-to-ground quantum communication network over 4600 km was implemented in China [10].

However, the unconditional security of the final key still might be broken because the imperfections of the practical devices could be exploited by Eve to bypass the security assumptions of QKD. For example, in the standard BB84 protocol, Alice is required to encode her information in the single-photon pulse. Nevertheless, instead of the single-photon source (SPS), the weak coherent source (WCS) that includes the multi-photon portion is widely used in most practical QKD systems. Then, Eve can perform the photon-number-splitting (PNS) attack by exploiting these multi-photon pulses [11,12]. So far, many quantum attack strategies have been discovered (see Table 1 in Section 5 for the detailed information, and Ref. [13] for a review).

In order to overcome the practical security threat, at least two solutions have been proposed. One is the new QKD protocol in which the loopholes of practical devices can be partially removed. For example, all loopholes in the detection part can be removed by the measurement-device-independent (MDI-) QKD protocol [14]. Moreover, by introducing Bell’s inequality [15,16], the unconditional security of device-independent (DI-) QKD can be proven with just a few basic assumptions. The other solution is security patching. The patches to certain known attacks are employed in a QKD system. By measuring or monitoring the parameters of the QKD system, the leaked information can be estimated. The security patching plays an important role to guarantee the security of a QKD system with imperfect devices. First, a security evaluation is necessary for most of the practical QKD system, even for MDI- and DI-QKD. Second, by monitoring the parameters of the QKD system, Alice and Bob can make sure that Eve cannot perform some quantum attacks, and then the performance of a QKD system can be improved.

In this paper, we review the development of security evaluation technology for QKD. Although there are many different QKD protocols based on both the discrete variables and the continuous variables, we focus our main attention on the decoy state BB84 protocol [17,18,19] here since it is the widely used protocol in many practical applications. In Section 2, we introduce the communication model of a typical QKD system, which can be divided into five modules (source, encoder, channel, decoder, and detector). Then, the basic security requirement for each module is introduced. In Section 3, by reviewing the main quantum hacking strategies in each module (The quantum channel is totally controlled by Eve, and the unconditional security of QKD is proven under the general coherent attack; thus the practical imperfections of the quantum channel only reduce the efficiency of the QKD system, but do not break its security.), it is clearly shown that, once some security requirements introduced in Section 2 are broken (due to the imperfection of the practical optical and electrical devices), the unconditional security of the final generated key will be compensated. In Section 4, we review the security model and show how to define the security parameter, which describes the deviation between the theoretical security requirement (introduced in Section 2) and the practical implementation (which could be exploited by Eve in Section 3). In Section 5, we introduce the security evaluation technology, and show the relationship between quantum hacking and security parameters.

## 2. Communication Model and Security Requirement

**Figure 1:**
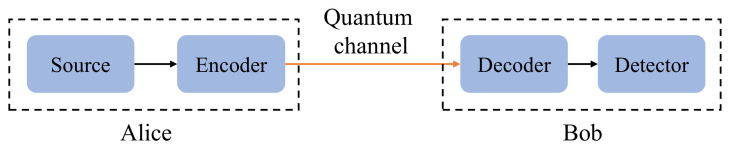
The concept communication model of a QKD system, which includes five modules: source, encoder, channel, decoder and detector. The source generates the required optical pulse, single photon pulse for BB84, or the weak coherent pulses with different average intensities. The encoder and decoder transform two classical bits into quantum states, back and forth. The detector absorbs the photon and registers the click of SPDs. The detailed definition and security requirement for each module are given in the main text.

According to a general communication model [20], a QKD system also can be divided as five parts (Figure 1): source, encoder, channel, decoder and detector. Now, we give the detail definition and security requirement of each module for a typical decoy state BB84 protocol.

***Source:*** In this module, a required optical pulse is generated, such as a single-photon pulse for the standard BB84 protocol. However, a perfect SPS is still unavailable for a practical QKD system, due to the complexity, stability, cost, and so on. Thus, for a practical decoy state BB84 protocol, the source generates a weak optical pulse with stable average intensity and known photon number distribution (PND). The most widely used source in a practical QKD system is the laser diode combining with an attenuator, which generates the weak coherent pulses following the Poisson distribution with an average intensity of μ≈0.1.

Although the security of QKD is compensated by the multi-photon pulse in the WCS, the decoy state method [17,18,19] can be used to estimate the contribution of the single photon pulse. In other words, with the help of the decoy state method, the laser diode combined with an attenuator can be considered a SPS with finite-generation efficiency (the contribution of the multi-photon pulse could be removed from the total gain and bit error).

In order to guarantee the security of a decoy state BB84 QKD system, at least three basic assumptions are required [17,18,19]: (1) the average intensity and the PND of the source should be exactly stable and known; (2) the phase of each optical pulse should be uniformly randomized from 0 to 2π; (3) the decoy states should be indistinguishable in any dimensions except for the average intensity.

***Encoder:*** In this module, Alice transforms her two random classical bits (one is called *basis bit* and the other one *information bit*) into the quantum state. Then, one of four encoded quantum states is randomly generated by modulating the photon emitted by the sources. The two classical bits should be generated by a true random number generator (TRNG), such as the quantum random number generator [21,22]. The transformation from classical bits to quantum states is performed by a modulator, which is the core part of the encoder module and should be carefully protected to remove the existence of Eve. In order to make sure that Eve cannot distinguish the encoded quantum state, at least three assumptions are required [23,24,25]: (1) Eve does not have any information about the random number used by Alice (the random number used by Alice should be random and secure); (2) the encoded quantum state should perfectly match the standard quantum state required by the BB84 protocol (perfect quantum state preparing phase); and (3) the encoded quantum state should not be distinguished in any dimensions, except for the encoded degree (no information is leaked from the side channel).

***Quantum channel:*** In this module, the quantum state of Alice is transmitted to Bob. The fiber and free space are two typical quantum channels for QKD (the security of the classical channel used for the post-processing and device calibration is not considered here). In the security model of QKD, the quantum channel is assumed to be totally controlled by Eve, who can perform any operation admitted by the quantum mechanics. Thus, there are no security requirements for the quantum channel. However, the loss and noise of the quantum channel should amplify the flaws of the source and encoder [23], then limit the final key rate. Thus, a quantum channel with lower loss and noise is always necessary to improve the performance of the practical QKD system.

***Decoder:*** In this module, by measuring the optical pulse coming from the quantum channel, Bob could transform the quantum state into two classical bits (also called *basis bit* and *information bit*) again. The *basis bit* could be actively chosen with a T-RNG or passively registered with a beam splitter. The *information bit* is registered according to the click of SPDs. Since the optical pulse measured by Bob is totally controlled by Eve, the click of SPDs is determined by three parts, the encoded state of Alice, the operation of Eve and the measurement of Bob. In other words, the decoder module can be considered a box with one input and four outputs (although, in some QKD systems, Bob actively chooses his basis, and there are only two outputs in the decoder, but, theoretically speaking, we can consider the two basis one by one). For each optical pulse going into the box, it will output from one of the four outputs (presenting the two classical bits). Therefore, the following assumptions are required for the decoder module [24,25]: (1) the basis of Bob should be random, which cannot be controlled or known by Eve; (2) for each basis, Eve cannot control the output of the decoder box by manipulating the parameters of each optical pulse, such as the time, wavelength, and so on; and (3) no optical or electrical signal is leaked to the quantum channel from the decoder module. Since the decoder is the most weak part of the QKD system, we give a detailed discussion about it here. The first two assumptions above mean that Eve cannot control the probability P(i|λ) (i=0,1,2,3), which is the conditional probability that a photon outputs from the *i*-port of the decoder box given the hidden variable parameter λ controlled by Eve. Here, we remark that both the two phases that Bob randomly chooses as his basis and analyses of Alice’s information bit are included in “ *Decoder*” in this review. The main advantage here is that a part of the imperfection of the SPDs can be included in the *basis bit* and *information bit*. For example, the SPD blinding attack [26] for a polarization-encoding QKD system can be considered such that Eve can set the probability P(i|λ) as $P\left(i|I,Pol.\right)=p\delta_{ik}$ for each optical pulse. Here, *I* (Pol.) is the intensity (polarization) of Eve’s optical pulse, *k* is the index of SPD that should click if Eve is absent, and *p* is the probability that a optical pulse should be detected by Bob when Eve is absent.

***Detector:*** In the detector module, Bob measures the decoded optical pulse with SPDs and registers which SPD clicks (according to the security analysis, if more than one SPD click, Bob should randomly register one). Based on the *Decoder* module above, four SPDs are required. For the QKD system with only two SPDs, another two virtual SPDs that have the same parameters as that of the two factual SPDs can be introduced. Then, two virtual SPDs are used to measure the optical pulse for one basis and two factual SPDs for the other basis.

Thus, for the detector module, the following assumptions are required [27]: (1) all the clicks of the detectors can be registered by Bob; (2) no active optical or electrical signal is leaked to Eve from the detector.

## 3. Quantum Hacking

In this section, we briefly introduce the quantum attacks to show that Eve can exploit the imperfections of the practical devices to break parts of the required security requirements in Section 2, then compensate for the unconditional security of the final generated key. Here we should remark that, most of these attacks can be removed by taking the security parameters into the security model or monitoring the security parameters to remove Eve’s attack. The security parameter and the evaluation technology are discussed in next two sections. The detailed definitions of these security parameters are discussed in Section 4, which characterize the deviation between the theoretical requirement and the practical implement. The relationship between the quantum hacking and the security parameters is discussed in Section 5.

### 3.1. Source

**Figure 2:**
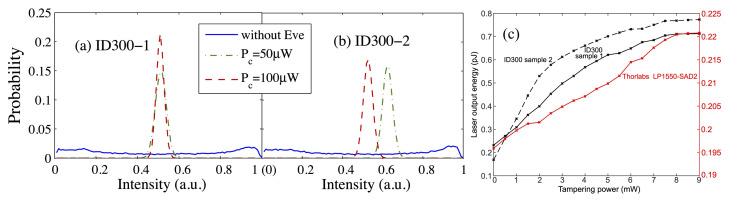
The phase distribution and intensity with and without Eve’s laser-injection attack, reprinted from Refs. [30,31]. (**a**,**b**) Phase distribution of Alice’s adjacent pulses tested from two samples of ID300 lasers. Without Eve’s attack, the phase is random. However, under 50 μW or 100 μW of Eve’s injected light, the phase follows a Gaussian distribution. (**c**) The increased intensity under laser-injection attack.

The phase randomization is a core assumption for a QKD with WCS. However, it has been shown that the phase might be unrandomized, due to imperfect implementation, which gives Eve a chance to distinguish the states and learn the secret keys [23]. Specifically, Eve can apply the unambiguous state discrimination (USD) measurement to distinguish decoy states and signal states if the phase is fully non-random [28]. With the help of homodyne detection, the encoded quantum state can be distinguishable when the phase of the source is just partially randomized [29]. Furthermore, the distribution of the phase can be tampered from uniform to Gaussian via the laser-injection attack [30] (see Figure 2a,b for detail).

**Figure 3:**
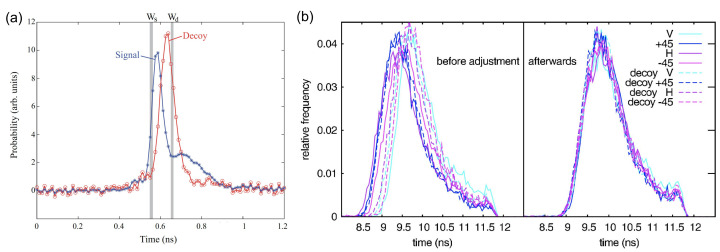
The experimental measurement of distinguishable states. (**a**) The pulse shapes of the decoy state and the signal state driven by electrical current. Reprinted from Ref. [32]. (**b**) Four encoded signal states and decoy states generated by individual laser diodes. Reprinted from Ref. [33].

The shape of the optical pulses is another type of vulnerability. If one drives the laser diode with different amounts of electrical current to generate decoy states and signal states, this driving mode may result in various lasering times and a lasting period for decoy states and signal states [32], as shown in Figure 3a. To exploit this loophole, Eve carefully chooses two observing windows, $W_{d}$ and $W_{s}$, to distinguish the signal state and decoy state [32] as shown in Figure 3a. The configuration of the multiple laser diodes may disclose the variation of the decoy states and signal states in the timing, spectral, and intensity degrees of freedom [33], which is shown in Figure 3b.

**Figure 4:**
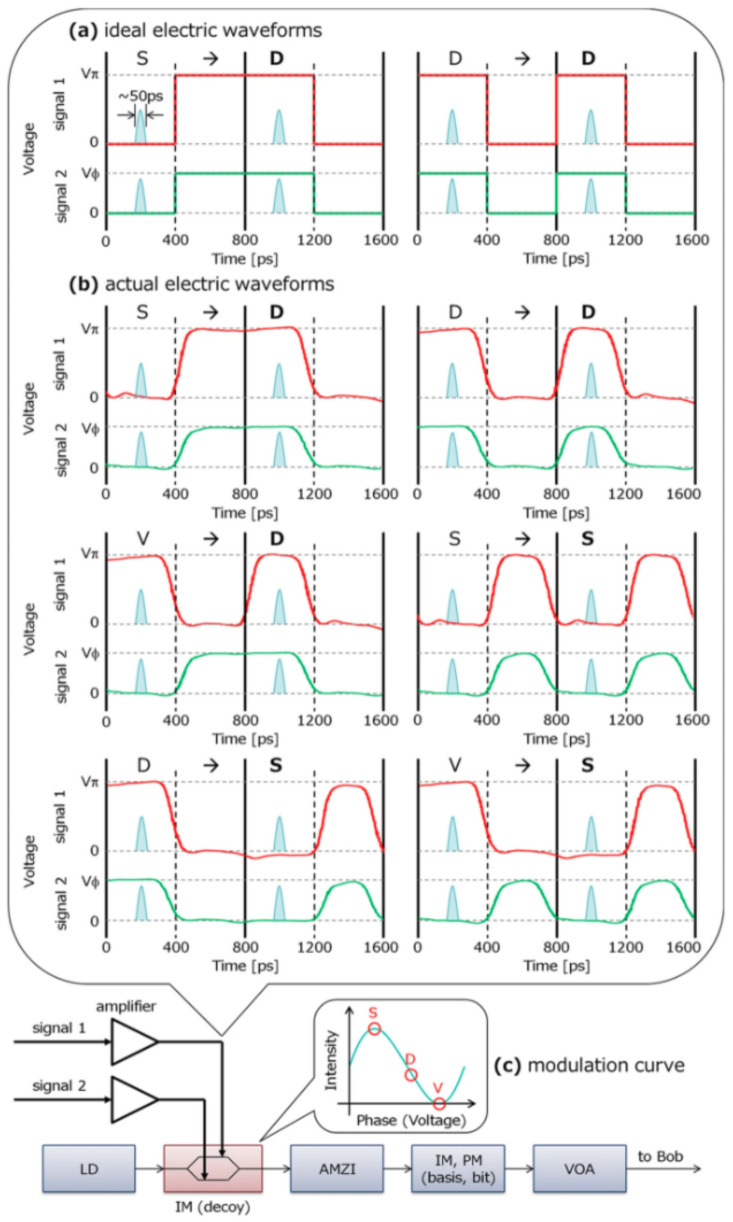
The typical testing result of intersymbol interference, which shows the intensity correlation between neighboring pulses. Reprinted from Ref. [34].

Time and spectrum are two other typical side channels. Intersymbol interference in time is usually disclosed in a high-speed QKD system [34]. The distorted driving signal for the intensity modulator may result in the intensity correlation between neighboring pulses in the time degree of freedom as shown in Figure 4, which breaks the assumption about independent and identical distribution. By actively shifting the arriving time of pulses to an intensity modulator, the spectrum of optical pulses can be correlated with the intensity of the light in a plug-and-play QKD system [35].

In the decoy-state BB84 protocol, the intensities of decoy states and signal states are preset to be optimal values, maximizing the key rate. However, these preset intensities might be manipulated by the laser-injection attack during the operating phase of a QKD system [30,31,36]. This is because Eve can lock Alice’s laser diode by injecting a bright light into it. As shown in Figure 2c, the intensity of Alice’s laser is increased to 3.07 times as the maximum with the raise of Eve’s injected power, which is not noticed by Alice and Bob. As a result, they may incorrectly estimate the contribution of the single photon pulse.

**Figure 5:**
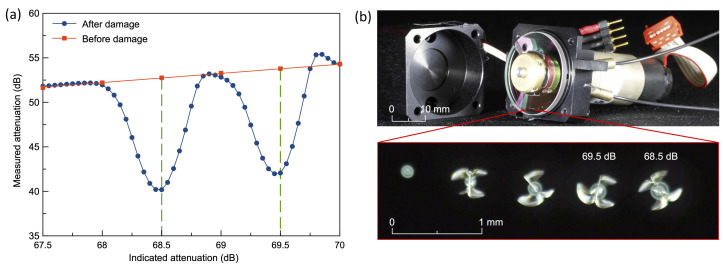
The testing results of the laser damage attack on attenuator. (**a**) The attenuation values before and after the laser damage attack. (**b**) The attenuator with the damaged areas. Reprinted from Ref. [38].

The intensity of Alice’s pulse also can be actively manipulated by Eve with the laser-damage attack on the optical attenuator [37,38]. Eve’s injected high-power light from the quantum channel first reaches the optical attenuator [39,40,41] and decreases the attenuation value [38]. Figure 5 illustrates the typical results of decreased attenuation after the attenuator being shined by 2.8 W laser for 10 s, which increases the intensity of Alice’s pulses.

### 3.2. Encoder

The encoder is always the target of Eve’s attack, since the quantum states is modulated here to represent the secret information. The security vulnerabilities of the encoder module come from both the encoding and non-encoding degrees of freedom.

For the encoding degrees of freedom, an imperfect encoder module may prepare non-orthogonal states. For example, in a phase-encoding QKD, the encoder is assumed to generate a state with one of four phases in $\left\{0,\frac{\pi }{2},\pi ,\frac{3\pi }{2}\right\}$. However, the actual phase modulated on the optical pulse may deviate from the required one, which allows Eve to partially distinguish the states [42]. Furthermore, the precision of modulation can be manipulated by modifying the arriving time of the pulses. For example, in a phase-encoding plug-and-play QKD system, Eve may remap the encoded phase of Alice by controlling the time that the optical pulse arrives at Alice’s modulator [43].

**Figure 6:**
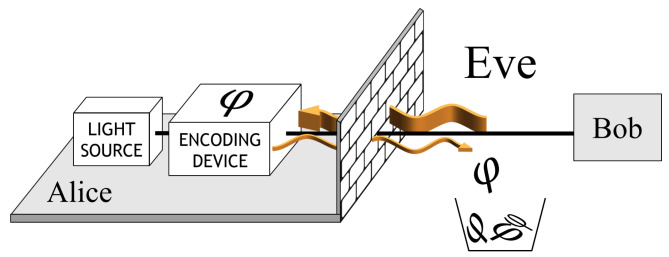
The working principle of Trojan horse attack. Reprinted from Ref. [44].

The non-encoding degrees of freedom also reveal side channels to Eve. For instant, in the Trojan horse attack [45], Eve actively sends optical pulses into Alice’s encoder from the quantum channel, a portion of which may be modulated by Alice and return to the channel again as shown in Figure 6. Since the reflected photon is measured by Eve and not transmitted to Bob, it does not increase the error rate and interrupt the QKD system. Therefore, Eve can silently learn the secret key.

It is notable that all the imperfections and attacks discussed in the source, Section 3.1, and the encoder, Section 3.2, not only affect the security of a decoy-state BB84 QKD system, but also may compromise the security of a MDI-QKD system that is immune to all attacks on the measurement unit. Since the MDI-QKD is out of the scope of this review, we will not discuss the security threat of it in detail here.

### 3.3. Decoder

At Bob’s side, the decoder module shall randomly choose the basis bit and the information bit as introduced in Section 2. In practice, these random choices may be known or controlled by Eve via the following attacks.

Regarding the basis bit, Bob may actively choose his basis with a modulator. Therefore, similar to the encoder, the choice of Bob’s basis may be eavesdropped by the Trojan horse attack on the modulator [46]. However, to reduce the probability that the Trojan horse light is detected by Bob’s SPDs, Eve may employ a hacking laser with a wavelength out of the SPDs’ sensitive range [47], which helps Eve hide her attack.

**Figure 7:**
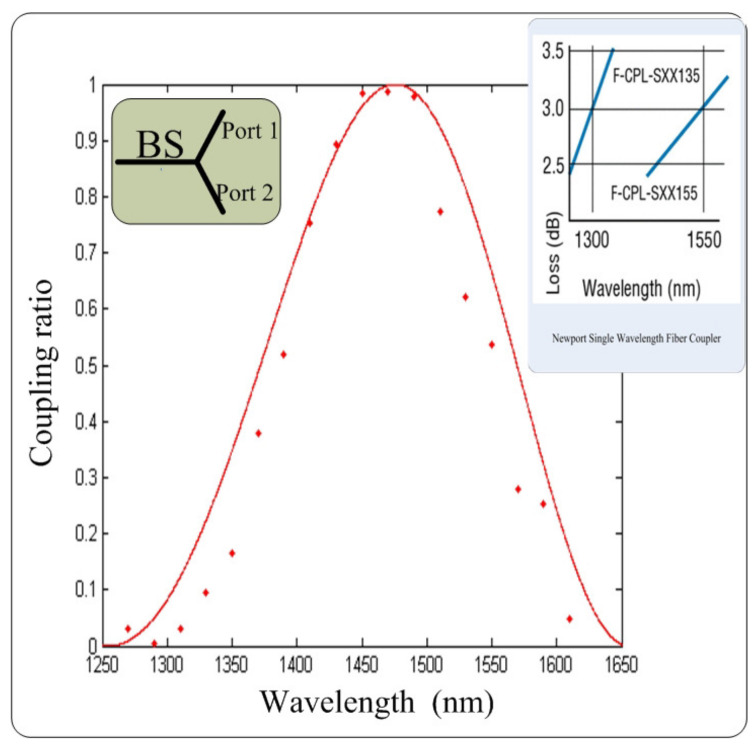
The change of coupling ratio depending on the wavelength, which can be exploited by Eve to conduct the wavelength-dependent attack. Reprinted from Ref. [48].

Another configuration of basis selection, named passive choice of measurement basis, is realized by a 50:50 beam splitter (BS). The randomness of the basis bit relays on the coupling ratio of the BS at the working wavelength, such as 1550 nm for a fiber-based QKD system. However, Eve may perform the wavelength-dependent attack [48]. Eve intercepts Alice’s state and resends a faked state whose wavelength depends on its basis. As shown in Figure 7, the different wavelengths may result in highly unbalanced coupling ratio of the BS, such as 99:1 or 1:99, which almost certainly determines the selection of the measurement basis.

The information bit is registered by the click from one of two Bob’s SPDs in the same basis. This result shall be fully determined by the randomness of Alice’s quantum state. However, in practice, Eve also can control the click of Bob’s SPDs, which breaks the randomness of the information bit (see Section 2 for the details). For example, Eve may exploit the loopholes of the SPDs to control the information bit. These types of attacks have been discovered the most so far, in which Eve tailors the arriving time, the intensity, the phase, or the polarization of the hacking pulses.

There are various types of attacks controlling the detection results by manipulating the arriving time of the hacking pulses, such as the time-shift attack [49], the efficiency mismatch attack [50,51], the dead-time attack [52], the after-gate attack [53], and the superlinearity attack [54].

**Figure 8:**
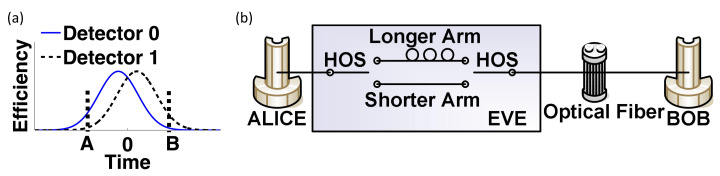
The working principle of time-shift attack. (**a**) The typical mismatched curves of detection efficiency. (**b**) The scheme of experimental demonstration. Reprinted from Ref. [49].

A typical detection efficiency curve is shown in Figure 8a, in which two detectors present a mismatch at point A and B. Then Eve can conduct the time-shift attack [49] by controlling the transmission delay of Alice’s pulse. Once the pulse passes through the shorter arm (Figure 8b) and arrives at moment A (Figure 8a), ‘”Detector 0” clicks with a higher probability than that of ‘”Detector 1”, and vice versa.

**Figure 9:**
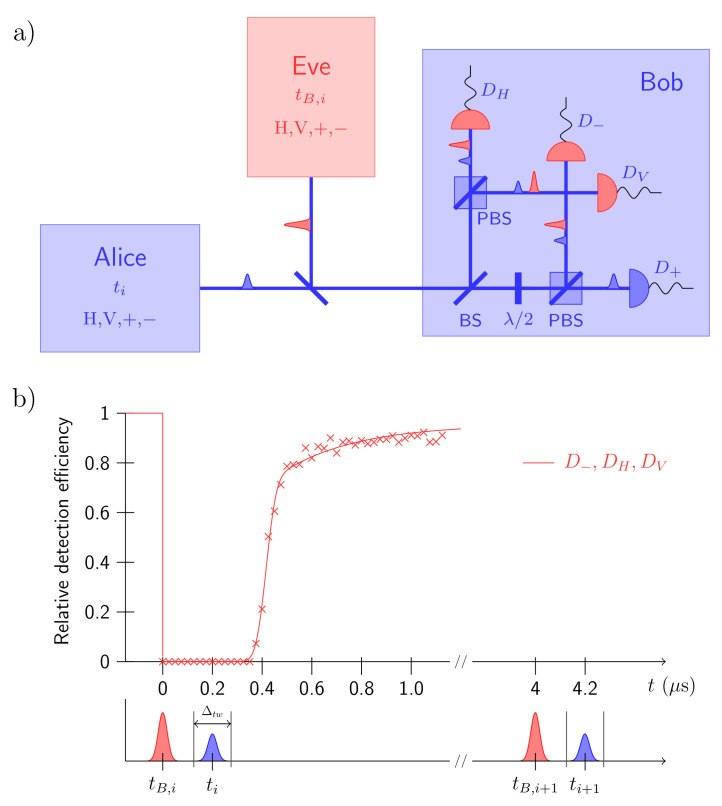
The illustration of dead-time attack. Reprinted from Ref. [52]. (**a**) The scheme of the dead-time attack; (**b**) the timings of faked pulses and signal pulses with detection efficiency of signal pulses under dead-time attack.

Another typical time-related attack is the dead-time attack [52]. Instead of tampering the signal state, Eve sends a faked state with multiple photons, for example |−〉 in Figure 9a, slightly before the signal state. The faked state triggers $D_{H}$, $D_{-}$, and $D_{V}$ click, following a period of dead time $\tau_{D}$, during which these three detectors are not sensitive to incoming photons. Only when the signal state of Alice is orthogonal to the faked state (|+〉), Bob registers a valid click on $D_{+}$. To avoid extra QBER, Eve’s faked state must be out of the detection time window $\Delta_{tw}$, while the signal state must be in the dead time period as shown in Figure 9b.

**Figure 10:**
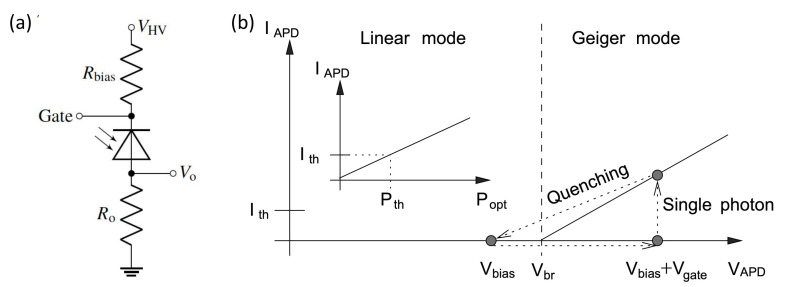
The illustration of the blinding attack. (**a**) The equivalent circuit related to the APD in a detector, reprinted from [56]; (**b**) the working modes of a APD, reprinted from Ref. [26].

By tailoring the intensity of the faked state, Eve also can control the information bit via the blinding attack [26,55,56]. Specifically, Eve first applies a strong continuous wave or pulsed light to transfer the SPD from the Geiger mode to the linear mode, then the SPD is no longer sensitive to a single photon. This is because, as shown in Figure 10a, the resistor $R_{b}ias$ reduces the voltage across the APD to be lower than the breakdown voltage ( Figure 10b), once a bright light illuminates at the APD. Then the blinded detector is employed in the “fake-state” attack. Eve intercepts Alice’s state and resends a faked state with a well-designed intensity to the blinded detector. The faked state triggers a click with high probability, even 100%, once Bob and Eve choose the same basis. Otherwise, Bob’s SPD almost does not click.

By increasing the power of the hacking light, Eve can conduct the laser damage attack to actively engineer multiple loopholes of a well-characterized detector [37]. A bright light with power 0.3 to 0.5 W can reduce the detection efficiency of the SPD by 80%–90%. This hacking light with a certain encoded state would permanently decrease the detection efficiency of a target SPD, which creates an efficiency mismatch between SPDs in Bob. Moreover, increasing the hacking power in the range from 1.2 to 1.7 W, the SPD is permanently blinded into the linear mode. Then, Eve performs the same as the blinding attack mentioned above, and the detector is fully controllable. In terms of the other power level, Eve may also change the characteristics of the detector, but there seems to be no help for Eve [37]. When the power of the hacking laser is over a threshold, 2 W in this case, the detector is catastrophically damaged.

### 3.4. Detector

The side channels of the detectors may leak the result of the detection, even though the decoder module randomly decodes the basis bit and the information bit. For example, the backflash attack takes advantage of the phenomenon that an APD has a chance to emit photons back to the channel after each detection [57]. The backflashed photon may be varied in the polarization, reflection time, and so on, depending on which SPD it comes from. Therefore, Eve can tell the clicked detector to learn the secret information. Another possible side channel in the detector is in the timing domain. Since the optical path to each detector or the response time of each detector may be slightly different, the registration time of detection might be varied depending on different detectors. If Eve has access to this timing side channel, she can derive the secret information [58].

## 4. Security Model and Parameters

According to the discussion above, Eve can break some security requirements and perform quantum hacking by exploiting the imperfections of practical devices. In this section, we show how to define the main security parameters in each module to describe the deviation between the theoretical requirement and the practical implementation. Before the main text, we give some discussions about the security parameter here. First, although the main security parameters are shown, the final key rate is not discussed in this paper. This is because it is still an open and very difficult question to calculate the final key rate by taking all the security parameters in one general security model. In some previous works [59,60], the flaws in the source and encoder were analyzed together, but most of flaws in the decoder and detector are still excluded. Second, these security parameters are measurable, and thus the legitimate parties can measure these parameters in the security evaluation phase, then evaluate the practical security and performance. In fact, by taking these security parameters into the key rate or monitoring them in real time, almost all of the discovered quantum hacking can be efficiently defeated.

### 4.1. Source

#### 4.1.1. The Intensity and Photon Number Distribution

Generally speaking, in order to estimate the contribution of the single-photon pulses, Alice should know the PND of her source $\left\{P_{n}\right\}$. However, the PND varies in the practical systems due to the fluctuation of the average intensity of the optical pulse [61], or Eve’s active attacks [30,31]. Thus, Alice should estimate the upper and lower bounds of the probability for each *n*-pulse, which is defined as

(1) $$P_{n}\in \left[P_{n}^{L},P_{n}^{U}\right].$$

Strictly speaking, Alice should measure the PND for the source with a photon number resolving detector. However, it is still quite experimentally challenging to achieve because only a few photons can be probably distinguished for some state-of-the-art detectors [62,63]. Thus, a reasonable assumption for Alice is that the source is a coherent state (any other source with a known PND in theory, such as the heralded single photon source [64], also can be analyzed with the same method given above) which is widely used in practical systems, and the variability of the PND can be estimated by the fluctuation of the average intensity of the source [38,61].

With the assumption given above, the deviation of the average intensity of the source is a proper parameter to bound the PND [61]. When Alice sends an optical pulse with average intensity μ, the factual intensity is bounded by

(2) $$\mu \in \{\mu_{L},\mu_{U}\}.$$

Then, Alice can redefine the average intensity of the optical pulses and the deviation of intensity, which are given by [61]

(3) $$\begin{array}{cc} \bar{\mu } & =(\mu_{U}+\mu_{L})/2,\\ \varepsilon_{\mu } & =\left|\mu_{U}-\bar{\mu }\right|. \end{array}$$

Thus, for the WCS, the bounds of the probability for each n− photon pulse are given by

(4) $$P_{n}^{L}=\frac{{\left(\bar{\mu }-\varepsilon_{\mu }\right)}^{n}}{n!}e^{-(\bar{\mu }-\varepsilon_{\mu })},\;P_{n}^{U}=\frac{{\left(\bar{\mu }+\varepsilon_{\mu }\right)}^{n}}{n!}e^{-(\bar{\mu }+\varepsilon_{\mu })}.$$

#### 4.1.2. The Random Phase of Source

In order to estimate the yield and error rate of the single photon pulses in the decoy state method, the source should be considered a mixed state of all photon number states. This assumption is valid only when the phase of the WCS is uniformly randomized within [0,2π]. Then the density matrix of the WCS can be written as

(5) $$\rho =\int_{0}^{2\pi }\frac{d\theta }{2\pi }|\sqrt{\mu }e^{i\theta }\rangle \langle \sqrt{\mu }e^{i\theta }|=e^{-\mu }\sum_{n=0}^{\infty }\frac{\mu^{n}}{n!}\left|n\rangle \langle n.\right|$$

Here, μ is the average intensity of the source, |n〉 is the Fock state with n− photon. Note that the security of BB84 also can be guaranteed with the discrete-phase-randomized WCS by modifying the post processing [65].

However, the phase-random assumption should be broken by Eve’s active attacks [28,29,66] as described in Section 3. Thus, the practical density matrices for each encoded state should be rewritten as

(6) $$\rho_{\alpha_{i}}=\int_{0}^{2\pi }d\theta P\left(\theta \right)|\alpha_{i}e^{i\theta }\rangle \langle \alpha_{i}e^{i\theta }|,$$

where α=z,x is the basis, i=0,1 is the bit for each basis, and P(θ) is the probability distribution of phase θ. The detailed expression of $|\alpha_{i}e^{i\theta }\rangle$ depends on the encoding of the QKD protocol. For example, $|\alpha_{i}e^{i\theta }\rangle =|\alpha e^{i\theta }\rangle$ for the polarization encoding, and $|\alpha_{i}e^{i\theta }\rangle =|\alpha e^{i(\theta +\varphi_{i})}\rangle_{s}{|\alpha e^{i\theta }\rangle }_{r}$ for the phase encoding. Here, $\varphi_{i}$ is the encoded phase, and the subscript s(r) means the signal (reference) pulse.

For the given state of Equation (6), the virtual entanglement states between Alice and Bob can be written as

(7) $$\begin{array}{c} \rho_{z}=\frac{1}{2}(|z_{0}\rangle \langle z_{0}|⊗\rho_{z_{0}}+|z_{1}\rangle \langle z_{1}|⊗\rho_{z_{1}}),\\ \rho_{x}=\frac{1}{2}(|x_{0}\rangle \langle x_{0}|⊗\rho_{x_{0}}+|x_{1}\rangle \langle x_{1}|⊗\rho_{x_{1}}). \end{array}$$

Here, $|z_{0(1)}\rangle$ and $|x_{0(1)}\rangle$ are the ideal quantum states required by the BB84 protocol. When the phase of the source is not uniformly randomized, the measured bit error in the *x*-basis does not equal the phase error in the *z*-basis. The phase error can be bounded by the measured bit error and the following parameter [23]

(8) $$\varepsilon_{RP}=\frac{1}{2}\left[1-F\left(\rho_{z},\rho_{x}\right)\right],$$

where F(ρ,σ) is the fidelity between ρ and σ.

#### 4.1.3. The Distinguishability of the Decoy States

For the discrete variable QKD with a non-single-photon source, the decoy state method [17,18,19] is considered one of the best ways to defeat photon-number-dependent attacks [11,12]. One of the basic assumptions for the decoy state method is that all the decoy states should be indistinguishable, except for the intensity. However, this assumption is hard to be guaranteed for some practical systems, due to the active attacks of Eve or passive side channels of Alice’s source [32,67].

When the side channels are taken into account, the density matrix of the decoy state with intensity $\mu_{i}$ can be written as

(9) $$\rho_{\mu_{i}}\left(\omega \right)\equiv \rho_{\mu_{i}}\left(t,\lambda ,w,\cdots \right),$$

where, ω includes all the side channels that can be exploited by Eve to distinguish the decoy states, such as time *t*, wavelength λ, waveform *w*, and so on. According to the analysis of Refs. [32,67], the distinguishability of the decoy states can be defined as

(10) $$\varepsilon_{DS}=\max_{i,j}\varepsilon_{DS}^{ij}\equiv \max_{i,j}\frac{1}{2}D\left(\rho_{\mu_{i}},\rho_{\mu_{j}}\right),$$

here, D(ρ,σ) is the trace distance of ρ and σ.

### 4.2. Encoder

#### 4.2.1. The Inaccuracy of the Encoded State

Due to the finite extinction ratio of practical optical devices or Eve’s active attacks [43], the practical encoded states of Alice may be different from the ideal states required by the QKD protocol. For example, Alice wants to send a quantum state |H〉, but the practical state sent by her may be cosθ|H〉+sinθ|V〉 with a small angle deviation θ≠0. The density matrix of the practical encoded state can be written as $\rho_{\alpha_{i}}^{en}$. Simply, if we assume that the encoded state of Alice is pure, then

(11) $$\rho_{\alpha_{i}}^{en}=P[\cos\theta_{\alpha_{1}}|\alpha_{0}\rangle +\sin\theta_{\alpha_{1}}|\alpha_{1}\rangle ]$$

where P[|a〉]=|a〉〈a| is the project operator. Then the deviation of the encoded state can be written as

(12) $$\varepsilon_{EN}=\max_{\alpha_{i},\beta_{j}}\varepsilon_{EN}^{\alpha_{i},\beta_{j}}=\max_{\alpha_{i},\beta_{j}}\frac{1}{2}\left[1-F\left(\rho_{\alpha_{i}}^{en},\rho_{\beta_{j}}^{en}\right)\right].$$

Here, we consider the worst case by maximizing $\varepsilon_{EN}^{\alpha_{i},\beta_{j}}$ for all α,β=x,z and i,j=0,1.

#### 4.2.2. The Side Channel of Encoder

The encoded states of Alice may be distinguishable in the non-encoded degrees of freedom, whose examples are given in Section 3. Then the practical density matrix of the encoded state should be written as

(13) $$\rho_{\alpha_{i}}^{si}=\left(\omega \right)=\rho_{\alpha_{i}}^{si}\left(t,\lambda ,w,\cdots \right)$$

where ω includes all the side channels that can be exploited by Eve to distinguish the encoded state. The distinguishability of the side channels can be defined as

(14) $$\varepsilon_{SI}=\max_{\alpha_{i},\beta_{j}}\varepsilon_{SI}^{\alpha_{i},\beta_{j}}=\max_{\alpha_{i},\beta_{j}}\frac{1}{2}\left[1-F(\rho_{\alpha_{i}}^{si},\rho_{\beta_{j}}^{si})\right]$$

In all the side channels, the Trojan horse attack plays an important role since it is one of the most well-known attacks in both classical and quantum communication. Here, we only consider the optical Trojan horse attack in QKD processing. When an optical pulse with intensity μ is reflected from Alice’s zone, the quantum state of such a Trojan horse photon can be written as

(15) $$|\sqrt{\mu_{\alpha_{i}}^{th}}\rangle ,$$

where the subscription $\alpha_{i}$ means the encoded state of Alice, and the superscription th means the Trojan horse pulse. We assume that the quantum state above is pure to maximize Eve’s information. Thus, the deviation of the Trojan horse photon belonging to each $\alpha_{i}$ can be defined as

(16) $$\varepsilon_{TH}=\max_{\alpha_{i},\beta_{j}}\varepsilon_{TH}^{\alpha_{i},\beta_{j}}=\max_{\alpha_{i},\beta_{j}}\frac{1}{2}\left[1-{\left|\langle \sqrt{\mu_{\alpha_{i}}^{th}}|\sqrt{\mu_{\beta_{j}}^{th}}\rangle \right|}^{2}\right]$$

### 4.3. Channel

In the security model of QKD, it is assumed that the channel is totally controlled by Eve who can do any operation and measurement admitted by the quantum mechanics. Thus, generally speaking, the imperfections of the quantum channel will not break the security of the generated key. However, the performance of the QKD system is compensated by the loss of the quantum channel. First, the final key rate is directly reduced by the loss and noise of the quantum channel. Second, the flaws of source could be amplified by the loss of the quantum channel [23].

For a quantum channel with transmittance η, the total count rate is the function of the loss, Q=Q(η). The deviation of source flaws ($\varepsilon_{EN}$, $\varepsilon_{SI}$, and $\varepsilon_{TH}$) should be rewritten as [23]

(17) $$\varepsilon_{\gamma }\to \varepsilon_{\gamma }/Q\left(\eta \right),$$

where γ=EN,SI,TH. Obviously, the deviation is large for long-distance communication. In order to overcome this problem, by introducing the “qubit” assumption, the loss-tolerant protocol was proposed by Tamaki et al. [68]. However, because of the side channels of the encoder [45] described in the next subsection, the “qubit” assumption is hard to be guaranteed in practical systems. Thus, the loss-tolerant protocol is not analyzed here.

### 4.4. Decoder

When the encoded states are flying into Bob’s zone, he randomly measures it with one of two bases. That is, the basis bit is randomly chosen by Bob (actively or passively). In each basis, the photon arrives at one of two SPDs to decide the value of Bob’s information bit. Strictly speaking, both the basis bit and the information bit should be totally random. However, due to the imperfection of the decoder, they could be controlled by Eve, such as the wavelength-dependent attack [48] and the detection efficiency mismatch attack [49] described in Section 3.

The weak randomness of Bob’s basis bit ($x_{0}$) and information bit ($x_{1}$) can be analyzed by introducing two hidden variables $\lambda_{0}^{de}$ and $\lambda_{1}^{de}$ [24,25]. By controlling $\lambda_{0}^{de}$ and $\lambda_{1}^{de}$, Eve can determine $x_{0}$ and $x_{1}$ for each pulse. Setting $k,k^{\prime }\in \left\{0,1\right\}$ as the value of $x_{0}$ and $x_{1}$, the probabilities that Bob obtains $x_{0}=k$ and $x_{1}=k^{\prime }$ are respectively given by

(18) $$\begin{array}{cc} p(x_{0}=k) & =\sum_{i}p\left(\lambda_{0}=i\right)p\left(x_{0}=k|\lambda_{0}=i\right)\\ p(x_{1}=k^{\prime }) & =\sum_{j}p\left(\lambda_{1}=j\right)p\left(x_{1}=k^{\prime }|\lambda_{1}=j\right), \end{array}$$

where $\sum_{i}p\left(\lambda_{0}=i\right)=\sum_{j}p\left(\lambda_{1}=j\right)=1$. $p(x_{0}=k|\lambda_{0}=i)$ is the conditional probability that Bob obtains $x_{0}=k$, given the hidden variable $\lambda_{0}=j$, and $p(x_{1}=k^{\prime }|\lambda_{1}=j)$ has the same definition. Obviously, Eve can determine the basis-bit and information-bit for each pulse by controlling the probability $p(\lambda_{0}=i)$ and $p(\lambda_{1}=j)$. Thus, the conditional probabilities $p(x_{0}=k|\lambda_{0}=i)$ and $p(x_{1}=k^{\prime }|\lambda_{1}=j)$ represent Bob’s basis bit and information bit leaked to Eve. In other words, the deviation of the decoder can be defined as [24,25]

(19) $$\begin{array}{c} \varepsilon_{DE}^{basis}=\max_{i}\varepsilon_{DE}^{basis,i}=\max_{i}\left|p(x_{0}=k|\lambda_{0}=i)-1/2\right|\\ \varepsilon_{DE}^{bit}=\max_{j}\varepsilon_{DE}^{bit,j}=\max_{j}\left|p(x_{1}=k^{\prime }|\lambda_{1}=j)-1/2\right|. \end{array}$$

Here we remark that in Equation (19), the deviation of basis bit ($x_{0}$) and information bit ($x_{1}$) are analyzed independently. However, generally speaking, Eve can control $x_{0}$ and $x_{1}$ at the same time with a joint hidden variable λ. Then Equation (19) should be rewritten as

(20) $$\varepsilon_{DE}=\max_{\lambda }\varepsilon_{DE}^{\lambda }=\max_{\lambda }\left|p(x_{0}=k,x_{1}=k^{\prime }|\lambda )-1/4\right|.$$

### 4.5. Detector

In the BB84 protocol, two or four SPDs are required by Bob to register the photon of Alice. There are two major imperfections for these SPDs. One is that the efficiency of these SPDs may depend on the parameters of the optical pulse, such as the time, wavelength, polarization, photon number (or intensity), and so on. The other one is the side channels, such as the reflection light [27,57,69].

For the first one, since each SPD represents the basis bit or information bit, it can be considered the flaw of the decoder (see Equation (19)). In this subsection, only the second one should be analyzed. The density matrix of the photon emitted into the quantum channel from Bob’s zone can be written as $\rho_{\alpha_{i}}^{Det}$. Then, Eve can guess which SPD clicks for each pulse by measuring the leakage signal. Thus, the deviation of the side channels can be defined as

(21) $$\varepsilon_{Det}=\max_{\alpha_{i},\beta_{j}}\varepsilon_{Det}^{\alpha_{i},\beta_{j}}=\max_{\alpha_{i},\beta_{j}}D\left(\rho_{\alpha_{i}}^{Det},\rho_{\beta_{j}}^{Det}\right),$$

where D(a,b) is the trance distance between *a* and *b*.

## 5. Security Evaluation and Standardization

The implementation of QKD systems, especially decoy-state BB84 ones, continues to mature. Commercial QKD products based on the decoy-state BB84 protocol are available in the market. Moreover, large-scale QKD networks all over the world are being deployed. During the commercialization and globalization of QKD, the reliability in use is essential for practical QKD systems, which highly depends on the security performance of the practical QKD system. However, as discussed in Section 3, the violation of the security requirement may be exploited by Eve to perform quantum hacking and then may threaten the practical security of a QKD system. In order to close the possible security loopholes (quantum attacks) and support the reliable use, one shall conduct the evaluation to verify the practical security of a QKD system. Generally speaking, in the evaluation phase, all the security parameters given in Section 4 should be carefully measured to guarantee that they are lower than the given threshold. Moreover, the optical and electrical signal also should be carefully monitored in the key-exchange phase to make sure that the evaluated security parameters are valid in practical situations. In other words, the evaluation phase provides the confidence to the QKD users and broadens the deployed range of QKD systems (if a QKD system passes through the evaluation test, it is secure even if there exist flaws).

To evaluate the security performance of a QKD system, the tester mimics as a quantum hacker to attack the QKD system under test, which may disclose the security vulnerabilities or show the defense against the attacks. For each testing item, the testing procedure follows the steps of conducting a certain quantum attack. Then, the corresponding behavior of the QKD system under attack shall be judged by a quantified criteria with a pass/failure threshold. For the decoy-state BB84 QKD system considered in this paper, most of the attacks described in Section 3 can be tested. Furthermore, the testing results can be quantified by the security parameters defined in Section 4.

The typical attacks and the corresponding security parameters are summarized in Table 1. According to Table 1, the attacks affecting the same security parameter in each module are classified, which indicates that fully characterizing a parameter requires multiple tests. The more tests are conducted, the better one knows about the practical performance of a QKD system. Generally, all the security parameters are considered in the final key rate. However, it is still a big challenge to take all of them into account in one security model at the same time.

This methodology of evaluation is possible to be standardized to serve as a third party certification for all decoy-state BB84 systems. The standardized verification provides a person-independent evaluation outcome, helping the customers build confidence and trust in QKD products. Most importantly, the security standard also guides the commercial company to produce the QKD products with high security performance, which promotes global deployment and enhances their application in various situations. The security evaluation standards are established by many organizations [70,71,72].

However, we should note that setting the thresholds for these security parameters is still an open question in practical application since a general security model including all the parameters is still unavailable; the final key rate may be rapidly reduced by parts of parameters, making the QKD system unusable. Therefore, a practical choice for the security evaluation and standard is to divide all the security parameters as two parts; one is considered in the security model (called *analyzed parameter*), and the other one is monitored (called *monitored parameter*). If a security parameter is analyzed in a security model, and some quantum hacking strategies by exploiting this loophole are discovered, this security parameter can be called an *analyzed parameter*. For these analyzed parameters, the QKD system is secure, no matter which threshold is set (the threshold only determine the final key rate). If a security parameter is not included in the security model, or no efficient hacking strategy is discovered by exploiting this loophole, this security parameter is called a *monitored parameter*. For these monitored parameters, the threshold should be carefully set to make sure that Eve’s potential attack can be removed within the current technology.

## Figures and Tables

**Table 1:** The main quantum attacks and security parameters.

Target	Attacks	Exploited Imperfections	Security Parameters
Source	Source attack [28,29]	Nonrandom phase	$\varepsilon_{RP}$
	Laser injection [30]	Nonrandom phase under laser injection	$\varepsilon_{RP}$
	Distinguishable decoy states [32]	Pump-current intensity modulation	$\varepsilon_{DS}$
	Side channels in free-space Alice [33]	Multiple laser diodes	$\varepsilon_{DS}$
	Intersymbol effect [34]	Intensity correlation between neighboring pulses	$\bar{\mu }$, $\varepsilon_{\mu }$
	Wavelength-selected [35]	Intensity-related change in wavelength	$\bar{\mu }$, $\varepsilon_{\mu }$
	Laser injection [31]	Increased intensity under laser injection	$\bar{\mu }$, $\varepsilon_{\mu }$
	Laser damage [38]	Reduced attenuation under high-power illumination	$\bar{\mu }$, $\varepsilon_{\mu }$
Encoder	Source flaw [42]	Inaccurate state modulation	$\varepsilon_{EN}$
	Phase remapping [43]	Incorrect encoding among transition edges of modulation	$\varepsilon_{EN}$
	Trojan horse [44,45]	Reflection photon from injected laser	$\varepsilon_{SI}$ ($\varepsilon_{TH}$)
Decoder	Trojan horse [46,47]	Reflection photon from injected laser	$\varepsilon_{DE}^{basis}$
	Phase remapping [43]	Incorrect decoding among transition edge of modulation	$\varepsilon_{DE}^{basis}$
	Wavelength-dependent [48]	Wavelength-dependent coupling ratio of BS	$\varepsilon_{DE}^{basis}$
	Time shift [49]	Mismatched detection efficiencies	$\varepsilon_{DE}^{bit}$
	Efficiency mismatch [50,51]	Mismatched detection efficiencies	$\varepsilon_{DE}^{bit}$
	Dead time [52]	Individual dead time of each detector	$\varepsilon_{DE}^{bit}$
	After gate [53]	Linear mode of SPD	$\varepsilon_{DE}^{bit}$
	Superlinearity attack [54]	Superlinear response of SPD among transition edges	$\varepsilon_{DE}^{bit}$
	Detector blinding [26,55,56]	Linear mode of SPD	$\varepsilon_{DE}^{bit}$
	Laser damage [37]	Mismatched detection efficiencies and linear mode of SPD	$\varepsilon_{DE}^{bit}$
Detector	Backflash attack [57]	Backward-transmitted photons	$\varepsilon_{Det}$
	Timing side channel [58]	Detector-related detection timing tag	$\varepsilon_{Det}$

## Data Availability

All the data are available by contracting the authors.

## Abbreviations

The following abbreviations are used in this manuscript:

QKD	Quantum key distribution
PND	Photon number distribution
PNS	Photon number splitting attack
SPS	Single photon source
SPD	Single photon detector
WCS	Weak coherent source
